# Reverse Transcription Recombinase-Aided Amplification Assay for Newcastle Disease Virus in Poultry

**DOI:** 10.3390/pathogens14090867

**Published:** 2025-09-01

**Authors:** Nahed Yehia, Ahmed Abd El Wahed, Ahmed Abd Elhalem Mohamed, Abdelsattar Arafa, Dalia Said, Mohamed A. Shalaby, Arianna Ceruti, Uwe Truyen, Rea Maja Kobialka

**Affiliations:** 1 Reference Laboratory for Veterinary Quality Control on Poultry Production, Animal Health Research Institute, Agriculture Research Center, Dokki, Giza 12618, Egypt; nahedyehia@gmail.com (N.Y.);; 2 Institute of Animal Hygiene and Veterinary Public Health, Leipzig University, 04109 Leipzig, Germany; 3 Department of Virology, Faculty of Veterinary Medicine, Cairo University, Cairo 12211, Egypt

**Keywords:** RT-RAA, NDV, rapid diagnostics, RT-PCR

## Abstract

Newcastle disease (ND) is a highly contagious and economically significant viral infection that affects poultry globally, with recurrent outbreaks occurring even among vaccinated flocks in Egypt. Caused by the Newcastle disease virus (NDV), the disease results in substantial losses due to high mortality rates, decreased productivity, and the imposition of trade restrictions. This study aimed to develop a rapid, sensitive, and field-deployable diagnostic assay based on real-time reverse transcription recombinase-aided amplification (RT-RAA) for the detection of all NDV genotypes in clinical avian specimens. Primers and an exo-probe were designed based on the most conserved region of the NDV matrix gene. After testing ten primer combinations, the pair NDV RAA-F1 and RAA-R5 demonstrated the highest sensitivity, detecting as low as 6.89 EID_50_/mL (95% CI). The RT-RAA assay showed excellent clinical sensitivity and specificity, with no cross-reactivity to other common respiratory pathogens such as avian influenza virus, infectious bronchitis virus, *Mycoplasma gallisepticum* or infectious laryngotracheitis virus. All 25 field samples that were tested positive by real-time RT-PCR, including those with high CT values (~35), were detected by RT-RAA in 2–11 min, indicating superior sensitivity and speed. The assay requires only basic equipment and can be performed under isothermal conditions, making it highly suitable for on-site detection in resource-limited or rural settings. The successful implementation of RT-RAA can improve NDV outbreak response, support timely vaccination strategies, and enhance disease control efforts. Overall, the assay presents a promising alternative to conventional diagnostic methods, contributing to the sustainability and productivity of the poultry sector in endemic regions.

## 1. Introduction

Newcastle disease (ND) is an endemic viral disease that continues to impact poultry production due to its persistent circulation among flocks, including those that have been vaccinated [1]. The causative agent, Avian Orthoavulavirus 1 (AOaV-1), commonly known as Newcastle Disease Virus (NDV), is a negative-sense, single-stranded RNA virus belonging to the family *Paramyxoviridae* and the order *Mononegavirales* [2]. The viral genome is approximately 15,200 base pairs in length and encodes six structural proteins: matrix protein (M), large RNA polymerase (L), fusion protein (F), phosphoprotein (P), nucleocapsid protein (N), and haemagglutinin–neuraminidase (HN) [3,4]. Phylogenetic analysis of the *F* gene sequences divides NDVs into two classes: Class I, consisting mostly of avirulent viruses found naturally in wild aquatic birds, with a single virulent isolate reported [5], and Class II, which includes viruses with greater genetic diversity and virulence, encompassing at least 20 genotypes (I–XXI, excluding recombinant genotype XV) that infect a wide range of domestic and wild birds [3,4]. Currently, the majority of NDV outbreaks worldwide are caused by Class II genotypes V, VI, and VII [6]. The most prevalent genotype, NDV-II, was first isolated in Egypt in 1948 [7]. However, since 2011, outbreaks involving a newly predominant genotype, NDV-GVII, characterized by a velogenic pathotype, have been frequently reported among vaccinated backyard and commercial poultry flocks across various Egyptian governorates, leading to significant economic losses [8,9,10,11]. The clinical signs of ND, particularly respiratory distress such as dyspnea, can be easily confused with other avian respiratory diseases including highly pathogenic avian influenza (H5N1), infectious laryngotracheitis, infectious bronchitis, and low pathogenic avian influenza [12,13,14]. Therefore, sensitive, specific, and practical diagnostic methods are essential for effective prevention and control.

Numerous diagnostic techniques have been developed globally, including serological assays such as hemagglutination-inhibition, agar gel diffusion, neutralization, immunofluorescence, immunohistochemistry, and ELISA. However, widespread vaccination complicates serological interpretations due to antibody presence in healthy birds [15]. Methods such as real-time RT-PCR and conventional RT-PCR provide sensitive detection but require sophisticated thermocycling equipment and technical expertise [16,17]. Thus, there is a critical need for rapid, simple, and field-deployable diagnostic methods for NDV.

Recombinase-aided amplification (RAA), previously referred to as recombinase polymerase amplification (RPA), is an emerging isothermal nucleic acid amplification technique that eliminates the need for costly thermal cyclers and DNA denaturation steps. Compared to conventional molecular diagnostic methods, RAA offers several advantages, including lower cost, faster turnaround time (typically yielding results within 15 min), and minimal equipment requirements. These features enable the development of portable testing platforms suitable for use outside of fully equipped laboratories. The RAA reaction is based on enzymes such as strand-displacing polymerase, recombinase, and single-stranded DNA-binding proteins, which allow the performance in iso-thermal temperature conditions with a rapid turnaround time, making it ideal for on-site testing [18,19,20]. RAA has successfully been applied to detect various pathogens including avian influenza A virus and infectious laryngotracheitis virus [21,22,23].

Due to high genetic variability among avian paramyxovirus type 1 (APMV-1) genomes [24,25,26], designing universal PCR primers capable of amplifying all APMV-1 isolates has been challenging [6]. Most prior assays targeted the *F* gene [6]. However, sequence analysis revealed a relatively conserved region near the 5′ end of the *M* gene across various isolates [26].

This study aims to develop a real-time reverse transcription recombinase-aided amplification (RT-RAA) assay for rapid detection of Newcastle Disease Virus using universal primer targeting the conserved *M* gene.

## 2. Materials and Methods

### 2.1. Development of NDV RT-RAA

#### 2.1.1. Reference Strain

The reference strain of Egyptian NDV viruses (NDV/Egy/CK/F291/2024, GenBank accession number PP516376.1) was used to produce the standard viral strain by computing the 50% embryo infective dose (EID_50_) using the Reed and Muench technique as previously published [27].

#### 2.1.2. Clinical Samples

Clinical samples consisted of tracheal swabs collected from chickens in Egypt during routine surveillance conducted by the Animal Health Research Institute. The birds exhibited respiratory symptoms—including coughing, sneezing, nasal discharge, and depression—and the farms reported a high mortality rate ranging from 40% to 70%.

#### 2.1.3. RNA Extraction

Viral RNA was extracted from clinical samples and allantoic fluid of reference strain cultivation using the QIAamp Viral RNA Mini Kit (Qiagen, Hilden, Germany) in accordance with the manufacturer’s instructions. Two hundred microliters of tracheal swab suspensions of phosphate-buffered saline (PBS) and allantoic fluid were used in the extraction procedure. The RNA was stored at −80 °C after being eluted in a final amount of 50 µL.

#### 2.1.4. NDV RT-RAA Primers and Exo-Probe

Two forward, five reverse, and one exo-probe were used to determine the primer combination that yielded the highest RT-RAA sensitivity (Table 1). They were created by analyzing the sequences of 70 NDV entire genomes, which represent NDV strains worldwide. The analysis was performed using the MegAlign software 11.0.13 (DNA Star, Inc., Madison, WI, USA) (see Supplementary Table S1). Targeting the *M* gene, all of the selected RAA primers and exo-probes were designed in accordance with the Twist Amp exo RT kits guide (Twist Dx, Cambridge, UK) and ordered from Tib MolBiol (Berlin, Germany). Various oligonucleotide combinations were screened to select the most sensitive RT-RAA assay.

#### 2.1.5. NDV RT-RAA

Following the manufacturer’s instructions, the NDV RT-RAA was carried out in a 50 µL reaction volume using RT-RAA kits (Jiangsu Qitian Gene Biotechnology, Wuxi, China). In short, the following component volumes were utilized to prepare a 50 µL reaction mixture: 2.1 µL of each primer (10 pmol), 0.6 µL of exo-probe (10 pmol) 2.5 µL of magnesium acetate (14 mM), 25 µL of 4× rehydration buffer (Twist Amp, Cambridge, UK), and 5 µL of template. Everything was added to the lid of the RT-RAA strips, which include a dried enzyme pellet. The tube was closed, spun, mixed, and spun again. In an ESE Quant tube scanner (Qiagen, Hilden, Germany), fluorescence measurements and heating were carried out for 20 min at 42 °C. The FAM fluorescence signal intensities were measured every 20 s.

#### 2.1.6. Real-Time RT-PCR

Real-time reverse transcriptase polymerase chain reaction (rRT-PCR) was performed using the QuantiTect Probe RT-PCR Kit (Qiagen, Inc., Valencia, CA, USA) following the manufacturer’s instructions, along with specific primers targeting the *M* gene of NDV as described by Wise et al. (2004) [17]. The reactions were carried out using a StepOnePlus Real-Time PCR System (Applied Biosystems, Thermo Fisher Scientific, Waltham, MA, USA).

### 2.2. Validation of NDV RT-RAA

#### 2.2.1. NDV RT-RAA Sensitivity

The analytical sensitivity of the RT-RAA assay was evaluated using a serial dilution range of extracted NDV EID_50_ from 5 log 10 EID_50_ to 1 EID_50_/mL in 3 repetitions. Clinical sensitivity and specificity were calculated using standard formula.

#### 2.2.2. NDV RT-RAA Specificity and Cross-Reactivity

Specificity was determined by screening 15 RT-PCR negative samples (oral swabs) from apparently healthy chickens. Cross-reactivity was evaluated using the extract of six pathogens that produce respiratory signs, including highly pathogenic avian influenza (A/chicken/Egypt/1273CA/2012 (H5N1), H7N1 A/GUANDONG/17SF300/17/15, A/chicken/Egypt/F53/2024 (H9N2)), infectious laryngotracheitis virus U76 (ILTV), infectious bronchitis virus IBV/Chicken/Egypt/RLQP/F148/2023 (IBV) (GD Lab., Holland which is regional of OIE lab), and *Mycoplasma gallisepticum* Dam1 (Cornell University Diagnostic lab., Ithaca, NY, USA).

#### 2.2.3. Clinical Performance of NDV RT-RAA

Using 25 tracheal swabs from field cases from chicken in Egypt, the clinical performance of the NDV RT-RAA assay was assessed in comparison to real-time RT-PCR. The RNA copies per sample varied from low cycle thresholds (CT 13) to high cycle thresholds (CT > 35).

### 2.3. Statistical Methods

Based on the first derivatives of the real-time fluorescence signal measurement, the RT-RAA TT was computed using the Tube Scanner Software 1070066 09/2011 (Qiagen, Lake Constance, Germany). The signal of the negative control served as the cut-off value and any amplification curve crossing this threshold within the assay runtime was considered positive. The previously published standard formula [28] was used to evaluate the clinical sensitivity and specificity of the RT-RAA.

Using RStudio version 1.3.1093 (RStudio, Boston, MA, USA), a probit regression was performed, and the limit of detection was calculated. The illustration was created using the gplot2 package (v3.3.3). PRISM (Graphpad Software Inc. version 6.0 Mac, San Diego, CA, USA) was used for the comparison of NDV RT-RAA TT and real-time RT-PCR CT values.

## 3. Results

### 3.1. Primer Selection

**Figure 1:**
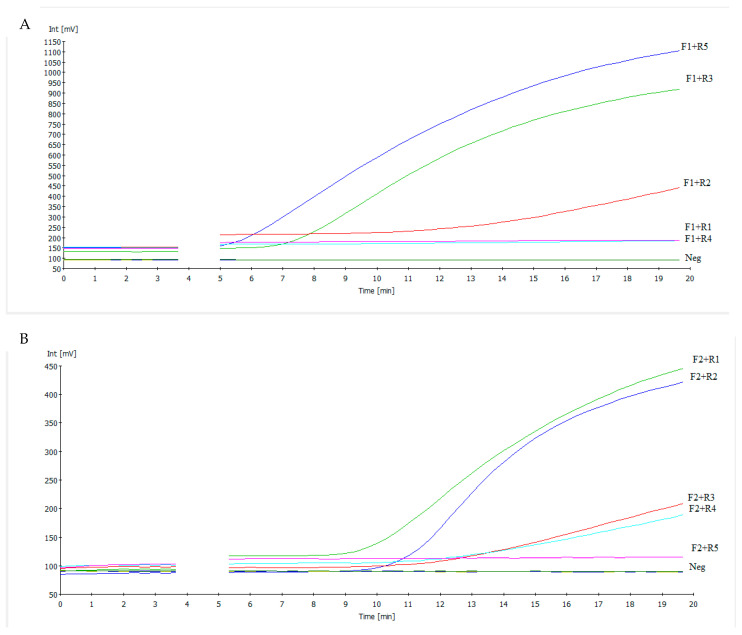
(**A**,**B**) The amplification curves of RT-RAA run using 10^5^ EID_50_/mL of the reference strain to test various primer combinations (F = forward primer, R = reverse primer). Molecular water was used as the negative control (Neg.). The drop in the fluorescence signal after three and a half minutes was due to the mixing step, which is necessary to produce a homogeneous RAA reaction. (**A**) Highest fluorescence of 1050 mV was achieved by the combination of primer F1 and reverse primer R5 with a time threshold value of 5.5.

To identify the most efficient primer combination capable of amplifying low copies of NDV RNA, 10 RAA primer combinations were evaluated using the extracted reference strain at a concentration of 10^5^ EID_50_/reaction (Figure 1). A negative control containing only molecular-grade water was included to detect any non-specific fluorescence signals. The time threshold (TT), measured in seconds, was defined as the point at which fluorescence intensity (in millivolts, mV) first rose above the baseline—established by the fluorescence level during the initial minute—based on first derivative analysis. The primer pair which performed best out of all the primer combinations with a TT value of 5.5 and a FAM value of 1050 was forward primer F1 and reverse primer R5 (Figure 1A). Consequently, this primer set (NDV RAA-F1+ NDV RAA-R5) was used for further assay validation.

### 3.2. NDV RT-RAA Assay Sensitivity and Specificity

**Figure 2:**
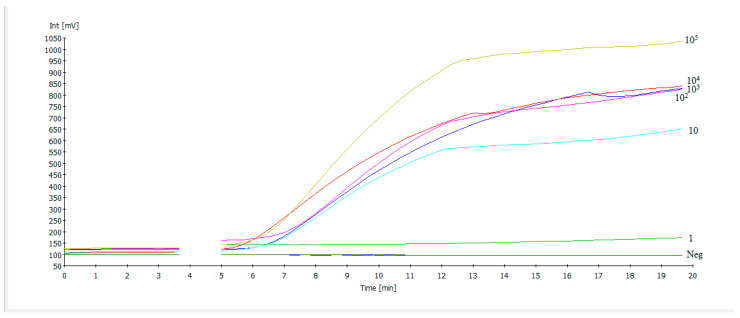
Amplification curves of the NDV RT-RAA using forward primer F1 and reverse primer R5 screening serial dilutions from 10^5^ EID_50_/mL to 1 EID_50_/mL of the reference strain to determine the analytical sensitivity. Dilutions down to 10 EID_50_/mL could be detected. Molecular water was used as the negative control (Neg.). The drop in the fluorescence signal after three and a half minutes was due to the mixing step, which is necessary to produce a homogeneous RAA reaction.

**Figure 3:**
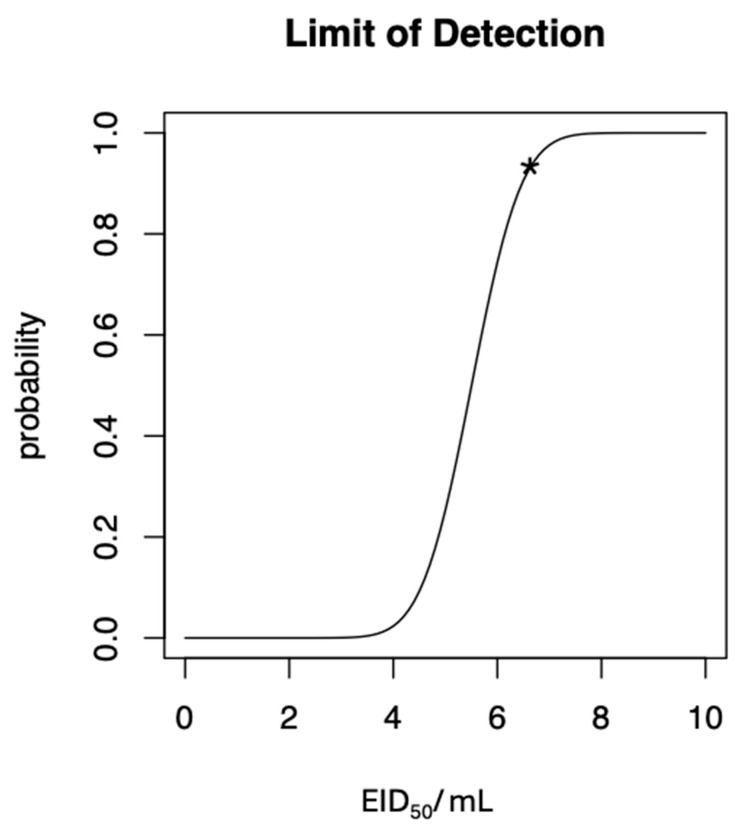
Probit analysis based on the results of eight NDV RT-RAA runs with 10^5^ to 1 EID_50_/mL of the reference strain. The limit of detection is 6.89 EID_50_/mL at a confidence interval 95% (95% CI) (depicted as asterisk).

A serial dilution range from 10^5^ to 1 EID_50_/mL of the NDV RNA standard was employed to determine the analytical sensitivity of the NDV RT-RAA test (Figure 2). Down to 10 EID_50_/mL were identified by primer F1 and R5. Probit analysis based on these results revealed a limit of detection of 6.89 EID_50_/mL (95% CI) (Figure 3). All 15 real-time RT-PCR negative samples (oral swabs) from apparently healthy chicken were also negative by RT-RAA.

### 3.3. NDV RT-RAA Cross-Reactivity

**Figure 4:**
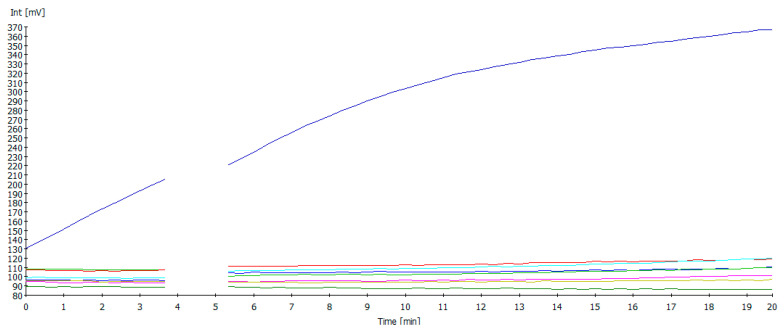
Amplification curves of the NDV RT-RAA for analytical specificity testing. Only the DNA of NDV was amplified (blue color), while there were negative results for H5N1, H7N1, H9N2, ILTV, IBV, and *Mycoplasma gallisepticum*. The drop in the fluorescence signal after three and a half minutes was due to the mixing step, which is necessary to produce a homogeneous RAA reaction.

No cross-reactivity was found screening the RNA/DNA of H5N1, H7N1, H9N2, ILTV, IBV, and *Mycoplasma gallisepticum* (Figure 4).

### 3.4. Clinical Performance of NDV RT-RAA Assay

**Figure 5:**
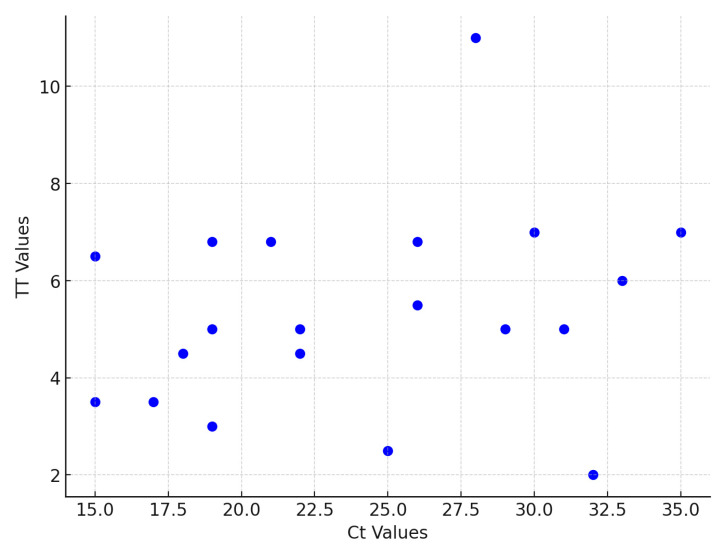
Comparison of time threshold (TT) values from RT-RAA assay and cycle thresholds (Ct) values from real-time RT-PCR of extracted clinical samples. Each sample is depicted as a blue dot. No correlation was found between the TT and the Ct values.

Extracts of 25 clinical samples were used to assess the clinical sensitivity of the RT-RAA assay. Samples with high and low virus loads could be identified by NDV RT-RAA in 2–11 min. This resulted in a sensitivity and specificity of 100%. The comparison of TT values and Ct values from real-time RT-PCR showed no correlation (Figure 5).

## 4. Discussion

ND is a highly contagious viral disease of poultry that can be economically devastating when caused by virulent strains, leading to high mortality rates and significant losses due to trade restrictions and control measures [29]. The disease remains a major concern worldwide, especially in endemic regions like Egypt. NDV strains are classified into Class I and Class II based on genetic characteristics, with Class II including the most virulent and diverse genotypes. Since 2011, Egypt has witnessed repeated outbreaks caused by the highly virulent NDV-GVII genotype, affecting both backyard and commercial flocks despite routine vaccination [8,9,10,11], underscoring the urgent need for improved control strategies and continuous monitoring.

The aim of this study was to develop a real-time reverse transcription RAA (RT-RAA) assay as a rapid, sensitive, and portable method for detecting all NDV genotypes in avian clinical samples. As the assay targets a highly conserved region within the *M* gene, it enables broad detection of both virulent and avirulent NDV strains, though it does not differentiate between pathotypes. A limit of detection of 6.89 EID_50_/mL and a clinical sensitivity and specificity of 100% were achieved.

Currently, RT-PCR and real-time RT-PCR are the most widely used methods for clinical detection of NDV due to their high specificity, low cost, and ability to perform absolute gene quantification [30,31]. However, these methods require expensive equipment, are time-consuming, and demand skilled personnel. Recombinase-aided amplification (RAA) is a novel isothermal nucleic acid amplification technique that eliminates the need for thermal cycling, simplifying instrumentation requirements. It can be performed using simple thermostatic devices, and the reagents, often provided as freeze-dried pellets, are stable during transport and storage without refrigeration, reducing costs and making RT-RAA ideal for on-site detection [32,33].

For sensitive amplification of NDV viral RNA standards, multiple forward and reverse primers and an exo-probe were designed to target this conserved *M* gene region. Out of 10 tested primer combinations, the pair NDV RAA-F1 and NDV RAA-R5 detected as low as 6.89 EID_50_/mL within few minutes, outperforming previous RT-RAA assays which required up to 30 min [34].

Mortality and respiratory signs like dyspnea can result from highly pathogenic avian influenza (H5N1), infectious laryngotracheitis, infectious bronchitis, and low pathogenic avian influenza and *Mycoplasma gallisepticum*—conditions with clinical symptoms similar to ND. This underlines the importance of specificity of the test to avoid misdiagnosis [12,13,14]. Our assay demonstrated a high specificity and no cross-reactivity with other diseases or negative swab samples. One reason for this might be the relatively long exo-probe and RAA primers enhancing target specificity [32].

All 25 chicken samples that tested positive by RT-PCR were detected immediately by the NDV RT-RAA assay. Remarkably, samples with high RT-PCR cycle threshold (CT) values (~35) were identified as positive by RT-RAA within 2–11 min, highlighting the assay’s high sensitivity. No direct correlation was found between RT-PCR CT values and RT-RAA threshold times (TT). This is not surprising because RAA amplification proceeds rather explosively, unlike the cyclic nature of real-time PCR [33].

The NDV detection has been previously attempted using RAA combined with lateral flow strips [34]. The advantage of this approach is the reduced equipment. However, it suffers from low sensitivity, reliance on visual interpretation, antibody dependency, sample volume inaccuracies, and high primer demands. In contrast, RT-RAA offers a portable, highly sensitive, and robust platform suitable for NDV detection at the farm level. Additionally, a reverse transcription RAA (RT-RAA) assay has been developed for the detection of NDV, utilizing fluorescence-based detection. Although this assay demonstrated good sensitivity and specificity, it still requires approximately 26 min to produce results and is based on amplification of the large polymerase (*L*) gene of NDV [34]. The *L* gene presents challenges for molecular diagnostics due to its large size, position as the last gene in the transcriptional gradient, and structural complexity [35]. Another study developed a reverse transcription loop-mediated isothermal amplification (RT-LAMP) assay for the detection of NDV, capable of detecting the virus within 35 min. A particular advantage of this approach is the establishment of a second assay targeting the *F* gene, which enables differentiation between virulent and avirulent strains [36].

Even though the developed assay is extremely quick, highly sensitive, and specific, it has some limitations. The extraction method used is not field-deployable, which may restrict its practical application in on-site testing scenarios. However, various rapid extraction techniques are now available that are adapted for field use [37,38]. The combination of rapid extraction of samples with the developed assay in a field setting should be evaluated in further studies. Furthermore, it is important to emphasize that respiratory diseases in poultry are often multifactorial, meaning that the detection of NDV alone may not definitively establish it as the primary causative agent without further investigation, especially because this assay does not distinguish between virulent and avirulent strains. Additionally, only a limited number of samples were analyzed in this study. Expanding the sample size in future investigations as well as including the vaccination status of the birds would strengthen the reliability and generalizability of the findings. In particular, testing of vaccinated birds should be included to assess potential cross-reactivity with vaccine strains.

## 5. Conclusions

The developed RT-RAA assay provides a rapid, accurate, and field-deployable alternative to conventional NDV diagnostic methods. Its isothermal nature and ability to detect viral RNA within minutes hold great promise for enhancing veterinary diagnostics, outbreak management, vaccination strategies, and ultimately sustaining the poultry industry against emerging and re-emerging NDV variants. Further research could refine assay design to differentiate vaccine strains from field isolates and enable multiplex detection formats with ability to differentiate virulent and avirulent strains.

## Figures and Tables

**Table 1:** Sequences of the exo-probe and primers used for NDV RT-RAA.

Name	Sequence
NDV-F1	5′AGAAAGTGACATTTGACAAGATAGAGGGAAAG 3′
NDV-F2	5′AGTGACATTTGACAAGATAGAGGGAAAGATAAG3′
NDV-R1	5′AGAGGCATTTGCTATAGGATAGCAGGCCGTC3′
NDV-R2	5′CTTATCTTTCCCTCTATCTTGTCAAATGTCACT3′
NDV-R3	5′CCTGAGGGGAGGCATTTGCTATAGGATAGCAG3′
NDV-R4	5′CAACCTGAGGGGAGGCATTTGCTATAGGA3′
NDV-R5	5′CCTGGGGAGAGGCATTTGCTATAGGATAG3′
NDV-exo-probe1	5′GCTCAGTGATGTGCTCGGACCCTCTG(BHQ1-dT)(THF) C (FAM-dT)TGTGAAGGCGAGAG-PH 3′

## Data Availability

The data presented in this study are available on request from the corresponding author.

## Supplementary Materials

The following supporting information can be downloaded at: https://www.mdpi.com/article/10.3390/pathogens14090867/s1, Table S1. NCBI accession numbers of all NDV strains analyzed to ensure primer and probe compatibility.

## Abbreviations

The following abbreviations are used in this manuscript:

APMV-1	Avian paramyxovirus type 1
AOaV-1	Avian Orthoavulavirus 1
BHQ	Black Hole Quencher
CI	Confidence interval
CT	Cycle threshold
EID_50_	50% embryo infective dose
ELISA	Enzyme-Linked Immunosorbent Assay
F	Fusion protein
FAM	Fluorescein Amidite
H5N1	Highly Pathogenic Avian Influenza subtype H5N1
H7N1	Highly Pathogenic Avian Influenza subtype H7N1
H9N2	Low Pathogenic Avian Influenza subtype H9N2
HN	Haemagglutinin-Neuraminidase
IBV	Infectious bronchitis virus
ILTV	Infectious laryngotracheitis virus
L	Large RNA polymerase
M	Matrix protein
mV	Millivolts
N	Nucleocapsid protein
ND	Newcastle disease
NDV	Newcastle Disease Virus
P	Phosphoprotein
PBS	Phosphate-buffered saline
RAA	Recombinase-aided amplification
RPA	Recombinase polymerase amplification
RT-PCR	Reverse transcription polymerase chain reaction
RT-RAA	Reverse transcription recombinase-aided amplification
THF	Tetrahydrofuran
TT	Time threshold
