# Supplementary material for: Reverse Transcription Recombinase-Aided Amplification Assay for Newcastle Disease Virus in Poultry
Source: Pathogens. 2025 Sep 1;14(9):867. doi: 10.3390/pathogens14090867 (PMC12473048; doi:10.3390/pathogens14090867)
Supplement: Supplementary file 1 [file pathogens-14-00867-s001.zip › pathogens-3782142-supplementary.pdf]

Article

Reverse Transcription Recombinase-Aided Amplification Assay for Newcastle Disease Virus in Poultry

Nahed Yehia [1], Ahmed Abd El Wahed [2], Ahmed Abd Elhalem Mohamed [1], Abdelsattar Arafa [1], Dalia Said [1], Mo-hamed A Shalaby [3], Arianna Ceruti [2], Uwe Truyen [2] and Rea Maja Kobialka [2] *

Supplementary

Table S1. NCBI accession numbers of all NDV strains analyzed to ensure primer and probe compatibility.

1	HQ697254	INDONESIA	COMPLETE GENOME
2	**HQ697255**	Indonesia	Complete genome
3	**MN557401**	Indonesia	Complete genome
4	**MN557402**	Indonesia	Complete genome
5	**MN557403**	Indonesia	Complete genome
6	**MN557404**	Indonesia	Complete genome
7	**MN557405**	Indonesia	Complete genome
8	**MN557406**	Indonesia	Complete genome
9	**MN557407**	Indonesia	Complete genome
10	**FJ754271**	China	Complete genome
11	**FJ754272**	China	Complete genome
12	**FJ754273**	China	Complete genome
13	**JX390609**	China	Complete genome
14	**JX193074**	China	Complete genome
15	**MK796808**	India	Complete genome
16	**MK796809**	India	Complete genome
17	**MK796810**	India	Complete genome
18	**KJ577585**	India	Complete genome
19	**JX316216**	India	Complete genome
20	**KP189357**	Russia	Complete genome
21	**KT962979**	Russia	Complete genome
22	**JN800306**	Peru	Complete genome
23	**JX974435**	Mexico	Complete genome
24	**KX822746**	Brazil	Complete genome
25	**KT948996**	Nigeria	Complete genome
26	**KU058680**	Nigeria	Complete genome
27	**MK674396**	UK	Complete genome
28	**MW342776**	Kenya	Complete genome
29	**MW342777**	Kenya	Complete genome
30	**MW342778**	Kenya	Complete genome
31	**MW342779**	Kenya	Complete genome
32	**MW342780**	Kenya	Complete genome
33	**MW342781**	Kenya	Complete genome
34	**MK495878**	Egypt	Complete genome; Egyptian LBM isolates
35	**MK495879**	Egypt	Complete genome; Egyptian LBM isolates
36	**MK495880**	Egypt	Complete genome; Egyptian LBM isolates
37	**MK495881**	Egypt	Complete genome; Egyptian LBM isolates
38	**MK495882**	Egypt	Complete genome; Egyptian LBM isolates
39	**MK495883**	Egypt	Complete genome; Egyptian LBM isolates
40	**MK495884**	Egypt	Complete genome; Egyptian LBM isolates

**41**	**MK495885**	Egypt	Complete genome; Egyptian LBM isolates
**42**	**MK495886**	Egypt	Complete genome; Egyptian LBM isolates
**43**	**MK495887**	Egypt	Complete genome; Egyptian LBM isolates
**44**	**MK495888**	Egypt	Complete genome; Egyptian LBM isolates
**45**	**MK495889**	Egypt	Complete genome; Egyptian LBM isolates
**46**	**MK495890**	Egypt	Complete genome; Egyptian LBM isolates
**47**	**MK495891**	Egypt	Complete genome; Egyptian LBM isolates
**48**	**MK495892**	Egypt	Complete genome; Egyptian LBM isolates
**49**	**MK495893**	Egypt	Complete genome; Egyptian LBM isolates
**50**	**MK495894**	Egypt	Complete genome; Egyptian LBM isolates
**51**	**MK495895**	Egypt	Complete genome; Egyptian LBM isolates
**52**	**MK495896**	Egypt	Complete genome; Egyptian LBM isolates
**53**	**MK495897**	Egypt	Complete genome; Egyptian LBM isolates
**54**	**MK495898**	Egypt	Complete genome; Egyptian LBM isolates
**55**	**MK495899**	Egypt	Complete genome; Egyptian LBM isolates
**56**	**MK495900**	Egypt	Complete genome; Egyptian LBM isolates
**57**	**MK495901**	Egypt	Complete genome; Egyptian LBM isolates
**58**	**MK495902**	Egypt	Complete genome; Egyptian LBM isolates
**59**	**MK495903**	Egypt	Complete genome; Egyptian LBM isolates
**60**	**MK495904**	Egypt	Complete genome; Egyptian LBM isolates
**61**	**MK495905**	Egypt	Complete genome; Egyptian LBM isolates
**62**	**MK495906**	Egypt	Complete genome; Egyptian LBM isolates
**63**	**MK495907**	Egypt	Complete genome; Egyptian LBM isolates
**64**	**MK495908**	Egypt	Complete genome; Egyptian LBM isolates
**65**	**MH614933**	Jordan	Genotype VIIi, complete genome
**66**	**MH044693**	Iran	PPMV-1, complete genome
**67**	**MZ712001**	Israel	Subgenotype VII.2, complete genome
**68**	**MZ712002**	Israel	Subgenotype VII.2, complete genome
**69**	**KJ920203**	Russia	PPMV-1; complete genome
**70**	**JQ013039**	Russia (Yakutiya)	Genotype Ib; complete genome
